# Supplementary material for: Muted extratropical low cloud seasonal cycle is closely linked to underestimated climate sensitivity in models
Source: Nat Commun. 2023 Sep 11;14:5586. doi: 10.1038/s41467-023-41360-0 (PMC10495370; doi:10.1038/s41467-023-41360-0)
Supplement: Supplementary file 1 — Supplementary Information [file 41467_2023_41360_MOESM1_ESM.pdf]

Supplementary Materials for

Muted extratropical low cloud seasonal cycle is closely linked to underestimated climate sensitivity in models

Xianan Jiang[1,2]*, Hui Su[1,3,4], Jonathan H. Jiang[2], J. David Neelin[4], Longtao Wu[2], Yoko Tsushima[5], and Gregory Elsaesser[6]

[1]Joint Institute for Regional Earth System Science and Engineering, University of California, Los Angeles, California, USA

[2]Jet Propulsion Laboratory, California Institute of Technology, Pasadena, California, USA

[3]Department of Civil and Environmental Engineering, Hong Kong University of Science and Technology, Hong Kong, China

[4]Department of Atmospheric and Oceanic Sciences, University of California, Los Angeles, CA, USA

[5]Met Office Hadley Centre, Exeter, UK

[6]NASA Goddard Institute for Space Studies, and Department of Applied Physics and Mathematics, Columbia University, New York, NY, USA

**Corresponding author address:* Xianan Jiang, Jet Propulsion Laboratory, California Institute of Technology, MS 233-300, 4800 Oak Grove Drive, Pasadena, CA 91109. Email: xianan@ucla.edu.

This file includes:

-Supplementary Tables 1-2

-Supplementary Figures 1-8

-Supplementary References

Supplementary Table 1: Analyzed CMIP6 GCMs and their predicted ECS*

Model Number	Model Name	ECS
1	ACCESS-ESM1-5	3.87
2 L	BCC-CSM2-MR	3.04
3 L	BCC-ESM1	3.26
4 L	CAMS-CSM1-0	2.29
5 H	CESM2	5.16
6 H	CESM2-FV2	5.14
7 H	CESM2-WACCM	4.75
8 H	CESM2-WACCM-FV2	4.79
9	CMCC-CM2-SR5	3.52
10 H	E3SM-1-0	5.32
11 L	FGOALS-f3-L	3.00
12 L	GISS-E2-1-G	2.72
13 L	GISS-E2-1-H	3.11
14 H	HadGEM3-GC31-LL	5.55
15 H	HadGEM3-GC31-MM	5.42
16	KACE-1-0-G	4.48
17	MCM-UA-1-0	3.65
18 L	MIROC6	2.61
19 L	MPI-ESM-1-2-HAM	2.96
20 L	MPI-ESM1-2-HR	2.98
21 L	MPI-ESM1-2-LR	3.00
22 L	MRI-ESM2-0	3.15
23 L	NorESM2-LM	2.54
24 L	NorESM2-MM	2.50
25	SAM0-UNICON	3.72
26 H	TaiESM1	5.34

* Labels "H" and "L" following the model number denote the high (> 4.75K) and low (< 3.3K) ECS models analyzed in this study. Predicted ECS from each model is adopted from[1], which is derived from the first 150 years that follow an instantaneous quadrupling of the atmospheric CO_2 concentration using a regression of the top-of-atmosphere net downward radiation versus the global mean near-surface air temperature[2]. Note that these ECS values are the "effective climate sensitivity", an approximation of the equilibrium climate sensitivity that might be biased[3,4].

Supplementary Table 2: Analyzed CMIP5 GCMs in Supplementary Fig. 7 and their predicted ECS[1]

Model Number	Model Name	ECS
1	ACCESS1-0	3.83
2	ACCESS1-3	3.53
3	bcc-csm1-1	2.83
4	BNU-ESM	3.92
5	CanESM2	3.69
6	CCSM4	2.94
7	CSIRO-Mk3-6-0	4.08
8	FGOALS-g2	3.38
9	GFDL-ESM2G	2.39
10	GFDL-ESM2M	2.44
11	GISS-E2-H	2.31
12	GISS-E2-R	2.11
13	IPSL-CM5A-LR	4.13
14	IPSL-CM5A-MR	4.12
15	inmcm4	2.08
16	MIROC-ESM	4.67
17	MIROC5	2.72
18	MPI-ESM-LR	3.63
19	MPI-ESM-MR	3.46
20	MRI-CGCM3	2.60
21	NorESM1-M	2.80

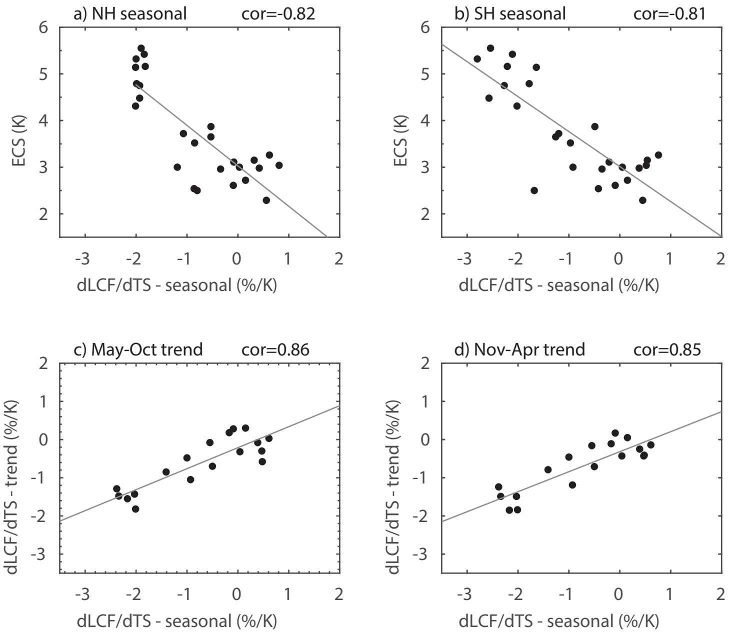

Supplementary Fig. 1 The equilibrium climate sensitivity (ECS) versus the dLCF/dTS associated with seasonal cycle and the long-term trend. (Panels a, b) Same as in Fig. 2b, but for Northern (a) and Southern (b) Hemisphere extra-tropics. (Panels c, d) Same as in Fig. 2d, but for dLCF/dTS associated with the long-term trend during May-Oct (c) and Nov-Apr (d).

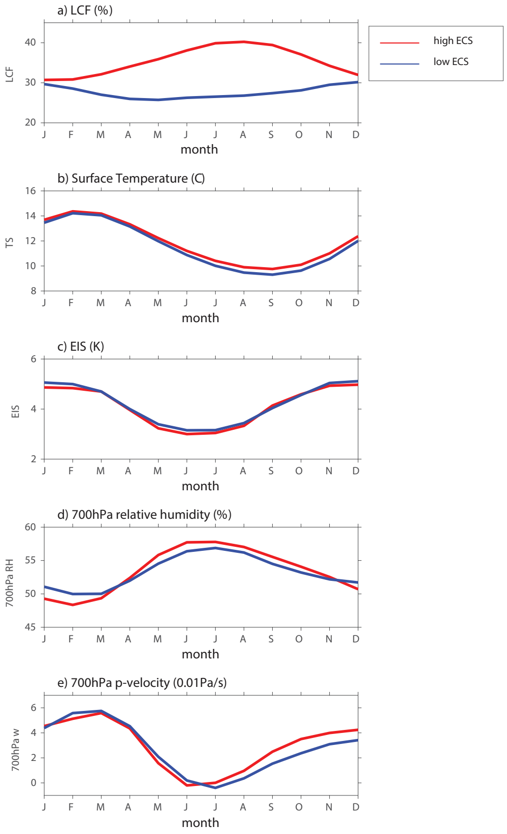

Supplementary Fig. 2 Cloud controlling factors associated with seasonal low-cloud fraction (LCF) variability. Seasonal cycle of LCF (a; Unit: %), surface temperature (b; Unit: K), the estimated inversion strength (c; Unit: K), relative humidity at 700hPa (d; Unit: %), and p-velocity at 700hPa (e; Unit: Pa s^{-1}) averaged over global longitudes between 60-30ºS for high (red) and low (blue) equilibrium climate sensitivity (ECS) composites. All these fields are derived based on climatological seasonal cycle from historical simulations during the period of 1980-2014.

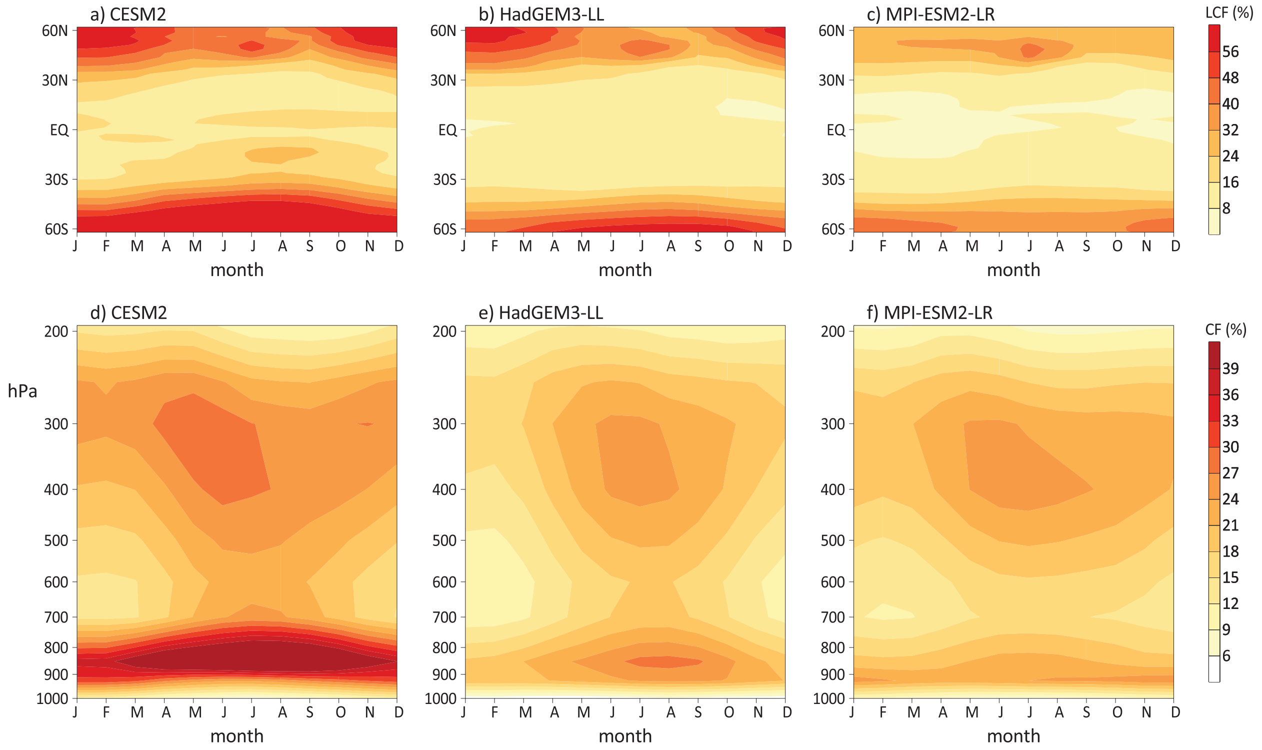

Supplementary Fig. 3 Seasonal variations of extratropical clouds in three selected models. Same as in Fig. 3, but for simulations from CESM2 (a,d), HadGEM3-LL (b, e), and MPI-ESM2-LR (c, f), respectively.

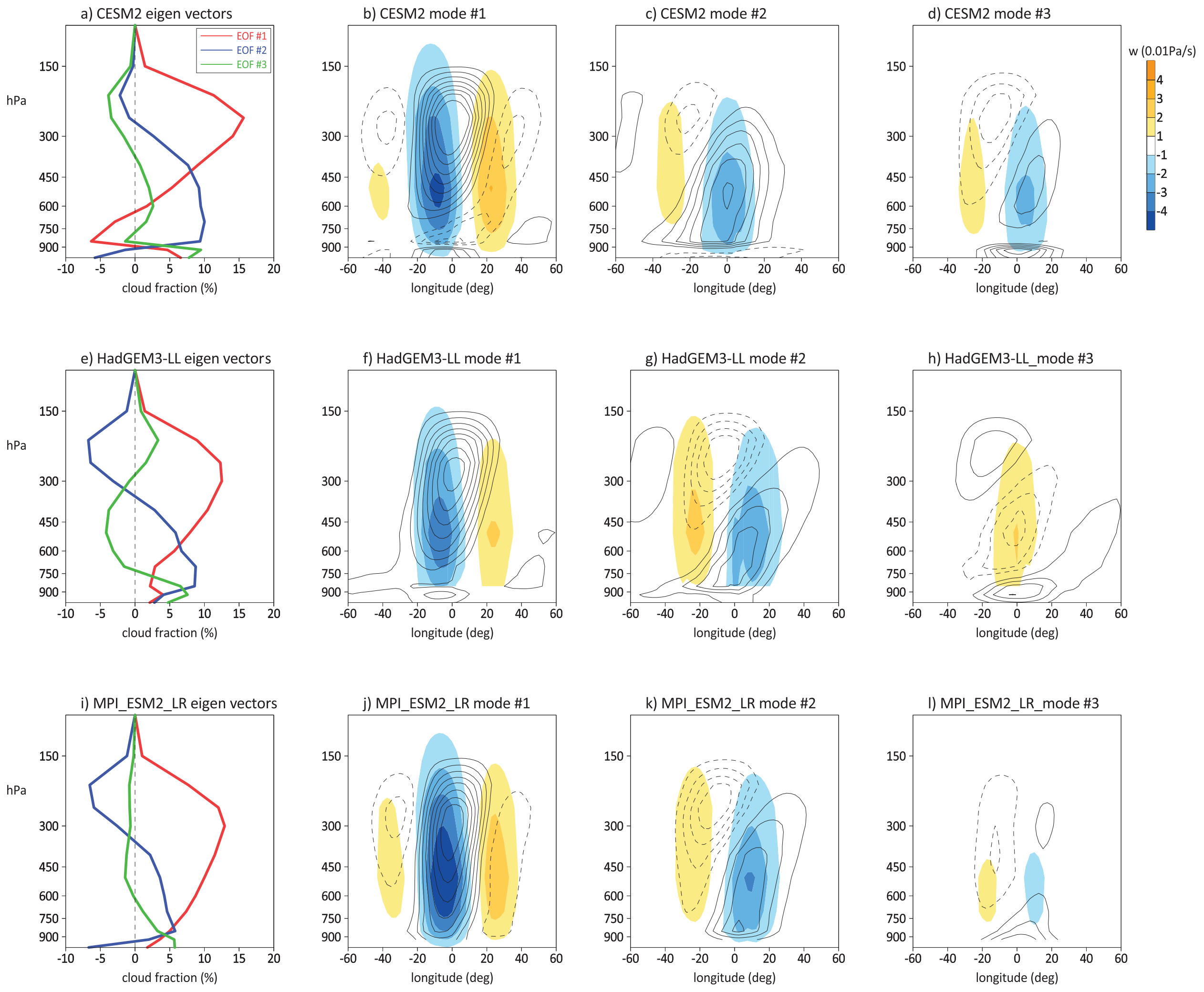

Supplementary Fig. 4 Leading cloud regimes in climate models. (Panels a, e, i) Eigenvectors of vertical cloud profiles associated with the three leading Empirical Orthogonal Function (EOF) modes (red: mode #1; blue: mode #2; green: mode #3) simulated in a) CESM2, e) HadGEM3-GC3-LL, and i) MPI-ESM2-LR. (Columns 2-4) Vertical-longitudinal profiles of anomalous p-vertical velocity (shading; see the color bar for scales with units of 0.01 Pa s^{-1}) and cloud fractions (contours with an interval of 1%, solid/dashed contours are for positive/negative values) associated with the three leading EOF modes in CESM2 (b-d), HadGEM3-LL (f-h), and MPI-ESM2-LR (j-l) simulations. See Methods for details in deriving the leading EOF modes and the vertical-longitudinal profiles of anomalous cloud fraction and vertical velocity associated with each EOF mode in different models.

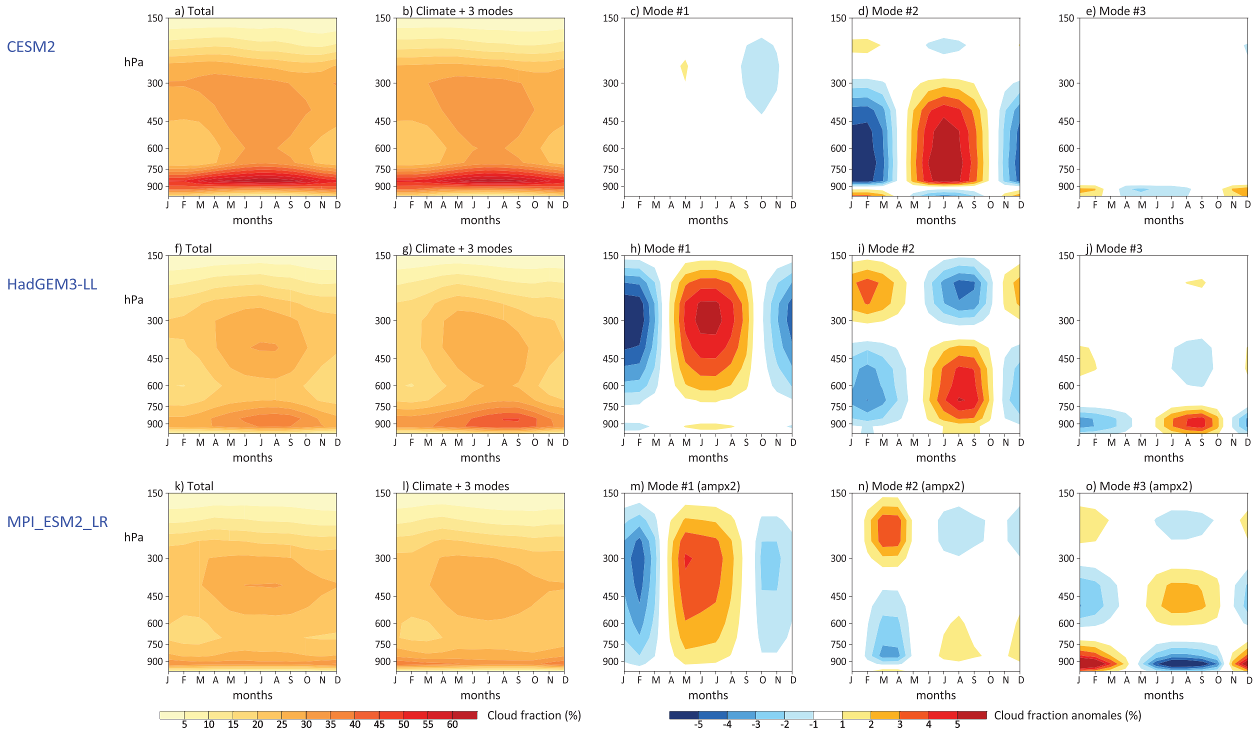

Supplementary Fig. 5 Reconstruction of model seasonal cloud variations using the leading cloud regimes. (Panels a-e) Climatological seasonal variations in vertical profiles of a) total cloud fractions, b) cloud fractions reconstructed using the climatological mean plus three leading Empirical Orthogonal Function (EOF) modes, and anomalous cloud fraction by c) the first, d) second, and e) third mode, respectively, based on CESM2. (Panels f-j) Same as the upper panels, but for HadGEM3-LL. (Panels k-o) Same as the upper panels, but for MPI-ESM2-LR. Note that amplitudes of anomalous cloud fractions in m)-o) have been increased by a factor of 2. All fields are averaged over extratropical Southern Oceans (55º-45ºS) for the period of 1980-2014.

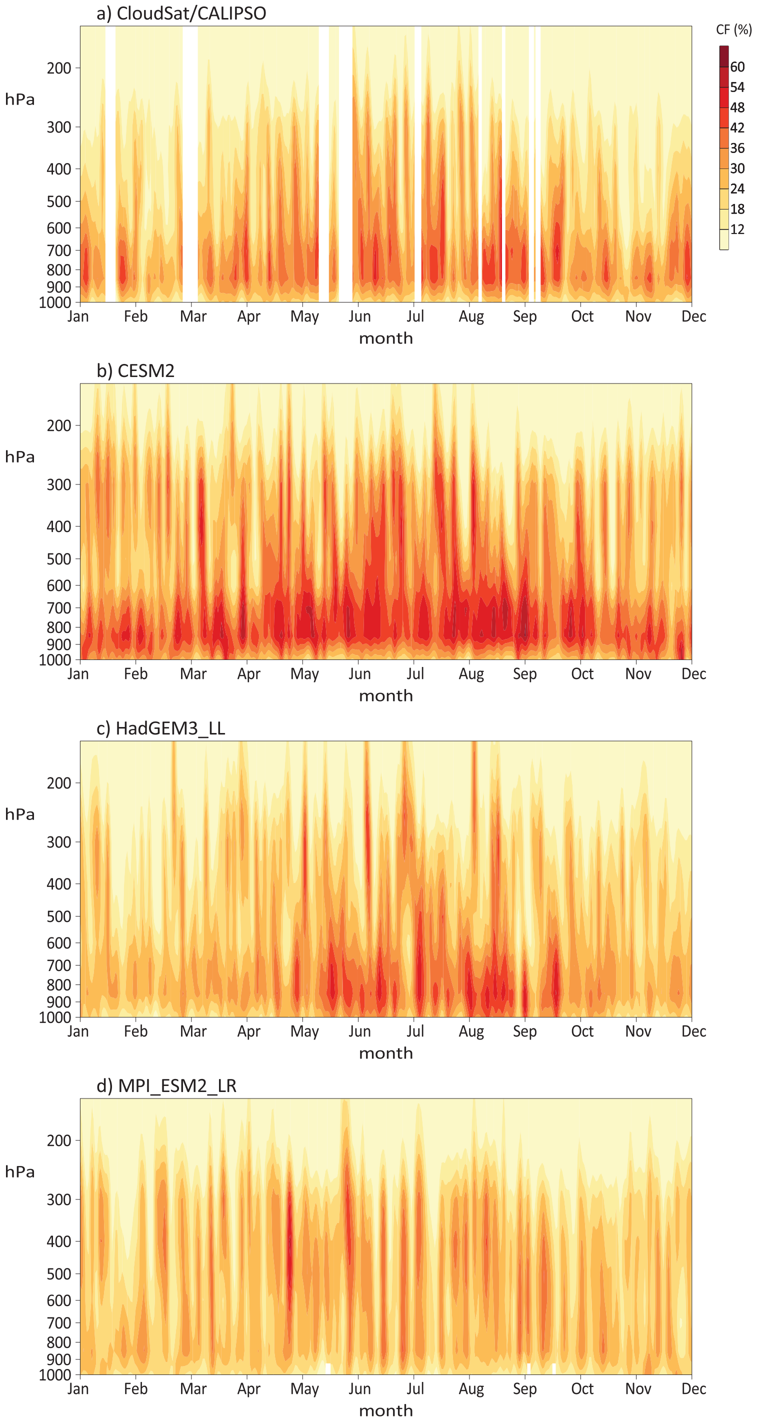

Supplementary Fig. 6 Snapshots of seasonal variations of vertical clouds over the Southern Oceans. Time evolution of daily vertical cloud fractions over a sub-region (75-100ºE; 55ºS-45ºS) during a randomly selected one-year period from a) the CloudSat/CALIPSO observations, and simulations from b) CESM2, c) HadGEM3-LL, and d) MPI-ESM2-LR. Units: %.

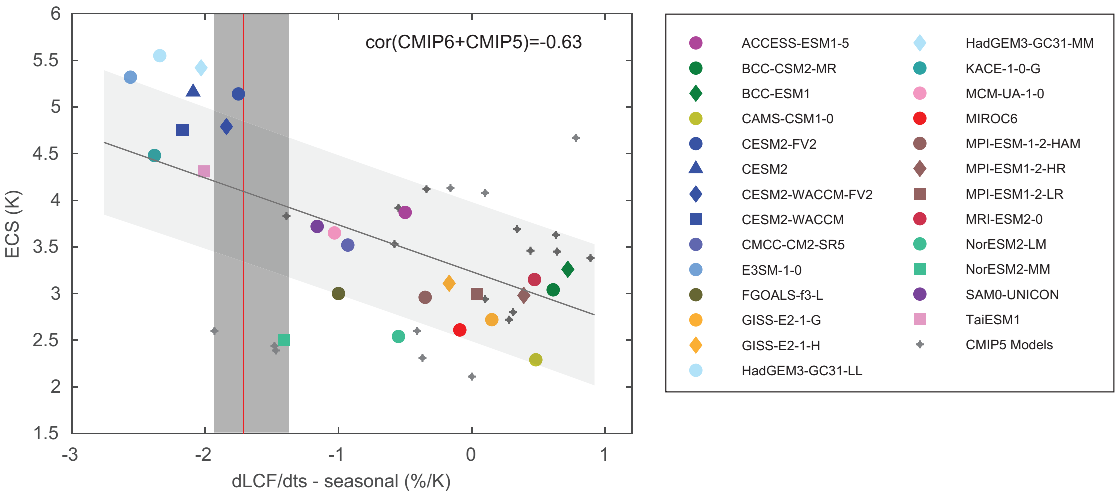

Supplementary Fig. 7 Seasonal dLCF/dTS versus the equilibrium climate sensitivity (ECS) in CMIP5 models. Same as in Figure 2b, but with simulations from 21 CMIP5 models included.

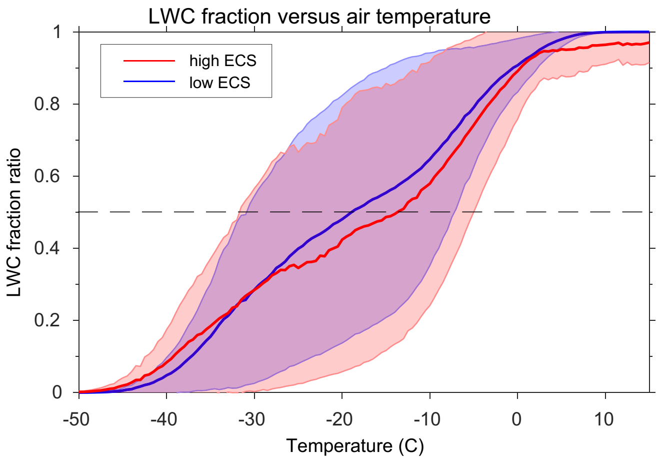

Supplementary Fig. 8 Liquid condensate fraction in the high and low equilibrium climate sensitivity (ECS) models. Composite liquid water condensate (LWC) fraction as a function of temperature from the high (red line) and low (blue line) ECS models. Warm and cold shading represent the spread of liquid condensation fraction denoted by one standard deviation among the high and low ECS models, respectively. The liquid condensate fraction is calculated by the ratio of cloud liquid water mass fraction to total cloud water (cloud liquid water + cloud ice water)[5,6]. All calculations are performed using monthly model output on vertical levels between 1000hPa and 10hPa over the extra-tropics (30-60°N/S) based on historical simulations from 1980-2014.

Supplementary References

1 Schlund, M., Lauer, A., Gentine, P., Sherwood, S. C. & Eyring, V. Emergent constraints on equilibrium climate sensitivity in CMIP5: do they hold for CMIP6? *Earth Syst. Dynam.* **11**, 1233-1258, doi:10.5194/esd-11-1233-2020 (2020).

2 Gregory, J. M. *et al.* A new method for diagnosing radiative forcing and climate sensitivity. *Geophys. Res. Lett.* **31**, doi:https://doi.org/10.1029/2003GL018747 (2004).

3 Rugenstein, M. *et al.* Equilibrium Climate Sensitivity Estimated by Equilibrating Climate Models. *Geophys. Res. Lett.* **47**, e2019GL083898, doi:https://doi.org/10.1029/2019GL083898 (2020).

4 Sanderson, B. M. & Rugenstein, M. Potential for bias in effective climate sensitivity from state-dependent energetic imbalance. *Earth Syst. Dynam.* **13**, 1715-1736, doi:10.5194/esd-13-1715-2022 (2022).

5 McCoy, D. T., Hartmann, D. L., Zelinka, M. D., Ceppi, P. & Grosvenor, D. P. Mixed-phase cloud physics and Southern Ocean cloud feedback in climate models. *Journal of Geophysical Research: Atmospheres* **120**, 9539-9554, doi:https://doi.org/10.1002/2015JD023603 (2015).

6 Zelinka, M. D. *et al.* Causes of Higher Climate Sensitivity in CMIP6 Models. *Geophys. Res. Lett.* **47**, e2019GL085782, doi:https://doi.org/10.1029/2019GL085782 (2020).
